# Sensor ToolKit (STK): Compact Multiplexing Potentiostat for Point-of-Care Applications

**DOI:** 10.1021/acs.analchem.5c07176

**Published:** 2026-02-17

**Authors:** Andrés Alberto Andreo Acosta, Alicia M. Maya, Simone Saporito, Moritz Kleinstraß, Jan Mitrovics, Pascal Blondeau, Francisco Javier Andrade

**Affiliations:** † Department of Analytical Chemistry, 16777 Universitat Rovira i Virgili, Carrer Marcel·lí Domingo, 1, 43007 Tarragona, Spain; ‡ JLM Innovation GmbH. Vor dem Kreuzberg 17, 72070 Tübingen, Germany

## Abstract

Embedded potentiostats have enabled the possibility of implementing electrochemical applications in a wide range of areas, from industry to healthcare. However, the optimization for either small size, low-cost devices, or sensor interfacing in existing platforms may lead to impractical outcomes. In this work, we propose Sensor ToolKit (STK), prototyped using off-the-shelf components and engineered to be a highly versatile, small, and low-cost platform of minimal complexity for the deployment of distributed IoT electrochemical sensors applications. The board implements an on-chip sequential input multiplexer that is especially suited for open-circuit potentiometry studies. The performance of the board was validated in potentiometric detection of potassium, hydrogen peroxide, glucose, and lactate, as well as self-current detection of hydrogen peroxide. Results show excellent analytical performance, comparable to that of benchtop instrumentation standards. The developed firmware and IoT framework enable the retrieval of sensor data wirelessly by a remote data server, which automates its real-time visualization using the Grafana web application, highlighting the potential of STK in distributed hyperconnected applications.

## Graphical abstract

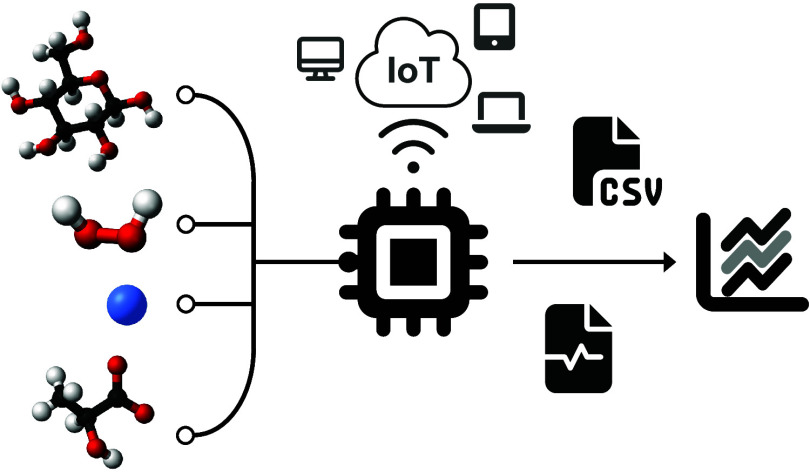

## Introduction

The paradigm shift toward personalized medicine and decentralized healthcare has created an unprecedented demand for real-time, remote monitoring of chemical and biochemical parameters across diverse environments. This transformation needs the widespread deployment of sophisticated yet accessible sensing platforms capable of delivering quality data in nonlaboratory settings.^1,2^ The integration of these tools into real-world applications presents unique engineering challenges. Factors such as device adaptability, environmental resilience, and seamless integration with existing workflows must be achieved for the successful deployment in distributed sensing networks. Design rules prioritizing miniaturization, cost, and ultralow power consumption have become fundamental prerequisites for mass adoption.^3–5^

Electrochemical techniques have emerged—in many applications—as the gold standard for developing portable analytical systems. This is due to their exceptional versatility across industrial process monitoring, environmental surveillance, healthcare diagnostics, and emerging Internet-of-Things (IoT) applications.^6–8^ With inherent advantages such as rapid response times, high selectivity, good sensitivity, and ease of miniaturization, electrochemical sensors have become a preferred transduction mechanism for point-of-care (PoC) diagnostics.^2^ For this reason there have been remarkable innovations in flexible electrochemical sensor architectures during the past decade. Miniaturized devices have been successfully integrated into diverse form factors including smart contact lenses for continuous glucose monitoring,^9^ wristband-mounted biosensors,^10^ epidermal patches for real-time sweat analysis,^11^ mouthguards for saliva biomarker detection,^12^ flexible sensors embedded into garments for tracking body functions,^13^ smart packaging for food safety monitoring,^14^ and even swallowable electronic pills for gastrointestinal tract monitoring.^15^ Despite these technological advances, widespread clinical and commercial adoption of these tools remains limited.

Traditional laboratory-grade electrochemical workstations and precision electrometers, while excelling in accuracy and measurement precision, fundamentally lack the portability, cost-effectiveness, and user-friendly interfaces required for widespread field deployment. These instruments hinder the widespread adoption of electrochemical sensing technologies since they show an elevated cost, require significant training for operation, and often display a large footprint. To address this critical gap, many attempts toward miniaturization, simplification, and cost reduction of instrumentation have been followed over the past decade. These efforts have been fueled by the proliferation of powerful, low-cost microcontrollers and comprehensive open-source software libraries, which have catalyzed the development of embedded potentiostats. This has enabled researchers to create custom analytical instruments tailored to specific applications.^16–20^

Current strategies to design miniaturized potentiostats generally follow two distinct approaches, each presenting unique advantages and limitations. In one of them, devices employ bottom-up construction using discrete electronic components.^20–23^ This approach offers maximum design flexibility and cost-effectiveness, but it demands extensive expertise in analog circuit design, signal processing, and sensor compatibility considerations. The resulting complexity often limits reproducibility and scalability, hindering widespread adoption beyond the reach of specialized research groups. Alternatively, a different path is the integration of analog front-ends (AFEs). This is a strategy that leverages commercially available components—including analog-to-digital converters (ADCs), digital-to-analog converters (DACs), and signal conditioning circuits—into single-chip solutions usually called AFEs. This AFE approach significantly reduces hardware-level design complexity and cost while maintaining analytical performance comparable to discrete implementations. Several research groups have demonstrated the viability of AFE-based potentiostats such as the LMP91000 (Texas Instruments, Dallas, TX)^18,24^ or the AD594x/ADuCM355 (Analog Devices, Inc., Wilmington, MA),^17,19,25^ enabling the development of low-cost, open-source platforms that have gained traction in academic and educational settings. Commercial PoC solutions are also available, including Zensor’s ECWP and EIWP series (Zensor R&D Co. Ltd., Taichung, Taiwan), MicruX ECSens (MicruX Technologies, Gijon, Spain), and Metrohm DropSens’ μSTAT handheld potentiostats series (Metrohm DropSens, S.L.U., Oviedo, Spain). Other AFE-based commercial implementations include the Sensit series (Palmsens BV, Houten, The Netherlands), based on the AD5940, or the Zimmer & Peacock platforms (Zimmer & Peacock AS, Horten, Norway), based on the LMP91000. Additionally, efforts to maximize the utilization of on-board microcontroller capabilities have shown promise, though external circuitry remains necessary to compensate for inherent ADC/DAC limitations and ensure proper sensor interfacing.^26,27^

Evidently, the intended application critically influences the potentiostat design requirements. Many applications for the point-of-need, while demanding portability and ease of use, may allow for larger form factors and more flexible operational parameters compared to wearable devices. In contrast, wearable biosensing platforms impose stringent constraints on device dimensions, weight, power consumption, and wireless connectivity protocols, necessitating fundamentally different design approaches.^2,4,28^ Connectivity solutions have evolved rapidly, encompassing USB serial interfaces for laboratory integration,^21,22^ Bluetooth for mobile device pairing and control,^29–31^ Wi-Fi for cloud-based data management,^18,19,32^ and emerging technologies including near-field communication (NFC)^33^ and long-range wide-area networks (LoRaWAN) for distributed sensing applications.^34^ However, many current implementations fail to fully address system-level integration challenges, resulting in solutions that compromise either analytical performance or practical deployment considerations.

Another critical capability for practical sensing applications is sensor multiplexing, since it enables simultaneous monitoring of multiple analytes or redundant measurements for enhanced reliability. While several research groups have demonstrated multiplexing implementations, most efforts have been limited to 2–4 channel configurations due to complexity constraints.^27,31^ Notable exceptions include 8-channel systems^35^ and even more ambitious 128-channel platforms,^36^ though these larger systems are typically designed for stationary PoC applications operated by trained technicians rather than compact wearable devices.^28,37^

The goal of this work is to address many of the identified gaps in portable electrochemical instrumentation by engineering a compact, versatile, and cost-effective IoT-enabled multiplexing platform specifically designed for electrochemical sensing applications outside of the laboratory. The Sensor ToolKit (STK) platform developed in this work leverages the advanced capabilities of the AD5941 potentiostat AFE (Analog Devices, Inc., Wilmington, MA), which has been selected for its industry-leading performance specifications and comprehensive functionality suite. There are multiple innovative features of this simple yet powerful device. First, the advanced switch-matrix architecture, which allows implementation of on-board multiplexing supporting up to 8 working electrodes with a shared reference electrode configuration, is optimized for space-constrained applications. Second, a dual-mode operation, supporting both open-circuit potential (OCP) and voltammetric modes, enables versatility across diverse sensing applications. Third, flexible connectivity options, with integration of both USB serial logging for laboratory uses and Wi-Fi connectivity to cloud server frameworks for remote monitoring applications. Last, but not least, IoT-ready architecture, with design optimization for distributed IoT deployments and healthcare applications. This can provide a foundation for next-generation wearable biosensing platforms.

## System Design

### Hardware Design

The Sensor ToolKit (STK) was designed using the AD5941 potentiostat analog front-end. This high-precision, low-power integrated circuit incorporates on-chip analog-to-digital (ADC) and digital-to-analog (DAC) converters designed to read signals and supply bias voltages as needed in most electrochemical-based portable applications. The ultralow-power DAC block comprises a 6-bit output, which sets the common voltage between the reference and working electrodes (V_ZERO0_), and a 12-bit output setting the bias voltage between the reference and counter electrodes (V_BIAS0_). We followed a zero-biased two-wire sensor approach with a switch configuration shorting RE and CE internally and a zero V_BIAS0_ being set between WE (also sense electrode, SE) and RE instead. Similarly, two transimpedance amplifier (TIA) circuits are present: a low-power TIA (LPTIA) and a high-speed TIA.

The implementation and operation of the different electrochemical techniques rely on the AD5941 sequencer block and the LPTIA, being able to measure DC signals below 3 mA. The 16-bit ADC can perform open-circuit potential (OCP) measurements with sampling rates ranging from 800 kHz in normal mode to 1.6 MHz in high power mode for a single channel. It also features a postprocessing block with several on-board signal filtering and processing options such as oversampling, averaging, and the possibility of computing statistical and discrete Fourier transform data. The AFE also includes a notch filter for the rejection of 50 and 60 Hz coupled noise coming from the mains supply. Enabling this postprocessing block or the on-board notch filter lowers the maximum sampling rate. For our purposes, the selection of the maximum oversampling rate supporting both 50 and 60 Hz notch filtering lowers the sampling rate to 300 Hz in normal mode for a single input channel. With the right configuration of the sequencer and internal switches, up to 8 differential input channels can be configured to be read sequentially sharing the same common point with a sampling rate of roughly 30 Hz per channel.

Electron transfer in electrochemical systems may occur with frequencies on the order of a few hundreds of kHz. However, most signals coming from electrochemical sensors are proven to be established well below few tens of Hz due to diffusion mechanisms limiting their intrinsic kinetics,^38,39^ thus proving that a sequential input multiplexing is feasible using the AD5941, avoiding the complexity of parallel multiplexing designs.^32^

AD5941 also features a high-speed 12-bit DAC that, combined with the high-speed TIA block, enables the measurement of wider bandwidth signals up to 200 kHz for voltammetric and electrochemical impedance applications. These have not been developed in this work. Amperometry applications are implemented for a single two-wire sensor measured via LPTIA. Functional block diagrams and schematics of the AD5941 can be found in the device datasheet.^40^

Table 1 summarizes the relevant technical specifications of the STK system and compares these values with those of other devices. A detailed comparison, including input/output characteristics, is provided in Table S1. The device can be powered via a 3.7 V conventional battery or the on-board USB-C port as needed. Figure 1 shows a photograph depicting the top side of the board, highlighting the main components. Pin headers are used for sensor interfacing via simple clamp–wire connections, as well as for expanding the on-board capabilities of the device with external circuitry.

**Table 1:** Performance Comparison of Sensor ToolKit with Other Potentiostat Devices.^a^

device	input CHs (on-board MUX)	sampling rate (Hz)^b^	resolution	power consumption (mW)	connectivity	largest dim. (mm)	cost (USD)
μSTAT 8000 ^41^	8 (8×WE vs 1×RE/CE)	1000	12 μV, 1 pA	n.d.	USB, Bluetooth	222	>5000
Sensit Wearable^30,42^	1 (2×WE)	1000	56 μV, 5.5 pA	92	USB, BLE 5.0	35	>1000
KickStat^24^	1 (−)	60	1 mV, 4.5 nA	n.d.	USB	22	n.d.
Hanitra et al.^31^	4 (2×OCP + 2×AMP)	300k	1.3 mV, 320 nA	150	USB, BLE 4.0	76	n.d.
Molderez et al.^36^	16 (128×WE)	MUX@651	n.d.	26.7 mW/ch	USB	163	80
SIC434x^33^	1 (−)	10	2.5 mV, 10 nA	*0*	NFC	85	>80
FreiStat^19^	1 (−)	300	54 nA	100^c^	USB, Wi-Fi	51	80
STK (this work)	1 (8×WE vs 1×RE/CE)	300, MUX@30	0.1 mV, 10 nA	250	USB, Wi-Fi^d^	53	50^e^

a CH: channel; MUX: multiplexer; BLE: Bluetooth Low-Energy; OCP: open-circuit potentiometric; AMP: amperometric; WE: working electrode; RE: reference electrode; CE: counter electrode; NFC: near-field communication.

b Typical value.

c Wi-Fi off.

d Easily expandable beyond BLE, NFC, Zigbee, Matter, and LoRaWAN.

e Production costs based on bill of materials. ∼USD 150 for a commercial product.

**Figure 1:**
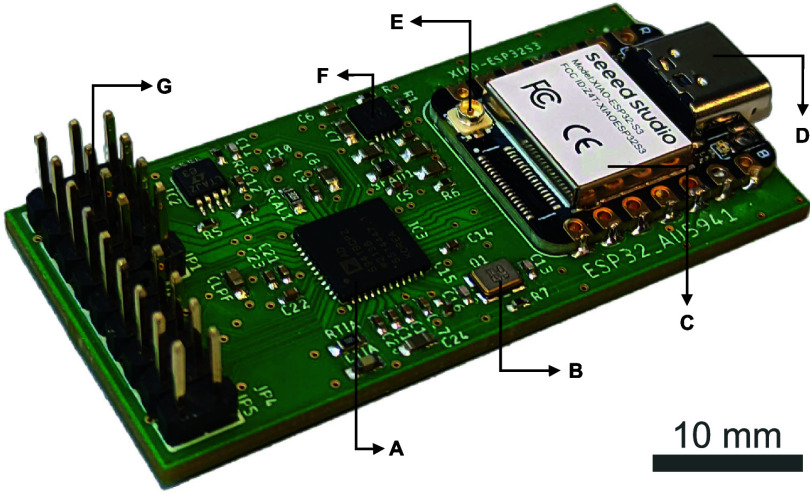
Photograph featuring the assembled STK board: (A) AD5941 analog front-end, (B) crystal oscillator, (C) microcontroller unit, (D) USB-C connector, (E) U.FL antenna connector, (F) low-dropout voltage regulator, and (G) input/bias and expansion pins.

With a size of only 25 × 53 mm, the STK board integrates the full capabilities of the AD5941 potentiostat AFE with a Seeed Studio’s XIAO form-factor socket (Seeed Technology Co. Ltd., Shenzhen, China). This unique design provides freedom in the selection of the microcontroller unit, leveraging a broad range of wireless hyperconnectivity capabilities such as Wi-Fi, Bluetooth Low-Energy (BLE), NFC, Zigbee, Matter, and LoRaWAN protocols. Moreover, the open hardware nature of this unit enables ready access to open-source software frameworks while also being Arduino-compatible. For our purposes, XIAO development kits built upon ESP32-S3, ESP32-C3 (Espressif Systems, Shanghai, China), and nRF52840 (Nordic Semiconductor, Trondheim, Norway) microcontrollers are implemented with Wi-Fi connectivity. Circuit design layout and schematics are shown in Figures S1–S3.

### Firmware and Server-Side Framework

The firmware for the Sensor ToolKit is aimed at porting the AD5940/AD5941 libraries provided by Analog Devices, Inc.^43^ and implementing the electrochemical techniques required for our applications. A general firmware was coded for any Arduino-compatible device. However, a deeper level of sophistication was achieved specifically for Espressif’s microcontroller units using Espressif’s official IoT development framework (ESP-IDF) and their open-source libraries instead. With the right implementation and interfacing, the code flashed to the microcontroller of the STK device can control the AD5941 through the serial peripheral interface (SPI) protocol and operate the electrochemical application of interest.

The self-hosted server side relies on the deployment and interoperation of open-source applications following the Docker container standard (Docker, Inc., Palo Alto, CA). Thus, firmware enables sending sensor readings via Wi-Fi to the data server using the message queuing telemetry transport (MQTT) protocol, as depicted in Figure 2, as well as generating data output as comma-separated values (CSV). Sensor data are then stored in the InfluxDB time-series database and automatically plotted using the Grafana web application. It can be retrieved by USB connection to a computer with any suitable serial communication tool or accessed remotely from the server side by any computer or smartphone connected to the Grafana app.

**Figure 2:**
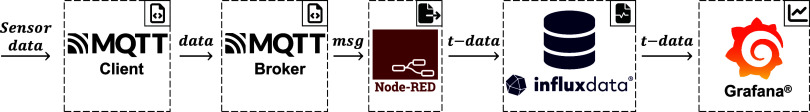
Overview of data flow from sensor to server side. Sensor data is sent via Wi-Fi to a remote server using the MQTT protocol, where it is stored in the InfluxDB database and plotted using Grafana. Application interoperation is managed by the Node-RED platform.

All the code used in this work, including library port for the microcontrollers, the implementation of the different electrochemical applications, and core server-side setup, can be found on GitHub.^44^

## Experimental Section

### Instrumentation and Methods

STK open-circuit potential measurements were validated against two benchtop laboratory equipment: a KEITHLEY 6514 digital electrometer (Tektronix Instruments, Inc., Malvern, PA) and a Lawson EMF 16 high input impedance interface (Lawson Labs, Inc., Malvern, PA). For the validation of amperometric measurements, the current was measured using the KEITHLEY 6514 electrometer. In all experiments, the STK board was powered via USB.

For measurements where both the STK and the electrometer were simultaneously used, the connection from the sensor was either parallel or periodically switched between the inputs to test the absence of crosstalk.

The local cloud server was deployed on a Raspberry Pi 5 (Raspberry Pi Ltd., Cambridge, U.K.) using the following Docker container images (Docker, Inc., Palo Alto, CA): Portainer CE (Portainer Pty Ltd., Sydney, Australia), Mosquitto (Eclipse Foundation, Ottawa, Canada), Node-RED (IBM, Armonk, NY), InfluxDB (Influx Data, Inc., San Francisco, CA), and Grafana (Grafana Labs, New York, NY). Firmware was developed using Espressif’s ESP-IDF (Espressif Systems, Shanghai, China) or Arduino integrated development environment (Arduino S.r.l., Monza, Italy).

### Sensor Preparation

The preparation of the paper-based ion-selective electrodes for the determination of potassium has been reported elsewhere.^45^ In essence, an ion-selective membrane cocktail was drop-cast on conductive paper properly shielded with a plastic mask. These electrodes were conditioned prior to their use. Measurements were performed using a double junction Ag/AgCl/KCl 3 M reference electrode (type 6.0726.100, Metrohm AG, Herisau, Switzerland) with a 1 M LiAcO bridge. For the detection of hydrogen peroxide, semiopen electrochemical cells (SOEC) were used, as described by Clua Estivill et al.^46^ Briefly, the SOEC comprises a vertically stacked design where two electrodes are overlapped, using a polyelectrolyte such as Nafion as an ionic bridge. Both the top electrode (working electrode) and the bottom electrode (reference electrode) are made of platinized sputtered paper coated with a Nafion layer. The determinations of glucose and lactate were performed by suitable functionalization of the SOEC with the corresponding oxidase enzyme. In all cases, electrodes were clamped to the STK input pins shown in Figure 1.

## Results and Discussion

### Performance Validation

#### Potentiometric Detection of K^+^

The Sensor ToolKit was first validated by measuring the open-circuit potential (OCP) of high impedance ion-selective electrodes (ISEs, 2–3 MΩ).^45^ The well-established potassium-selective electrode was selected to illustrate the detection of ions routinely performed in a clinical laboratory. Figure 3A displays the time trace of K^+^-selective sensors upon additions of different analyte concentrations. To demonstrate the performance of STK, the potentiometric response was recorded simultaneously with a digital electrometer and compared in real time. Analytical performance of the sensors is in good agreement with previous reports and a close-to-Nernstian sensitivity of 50.5 ± 0.8 mV/dec was achieved from 0.1 to 10 mM of K^+^, with a maximum offset mismatch of 2 mV and similar signal-to-noise ratios (Figure 3B).

**Figure 3:**
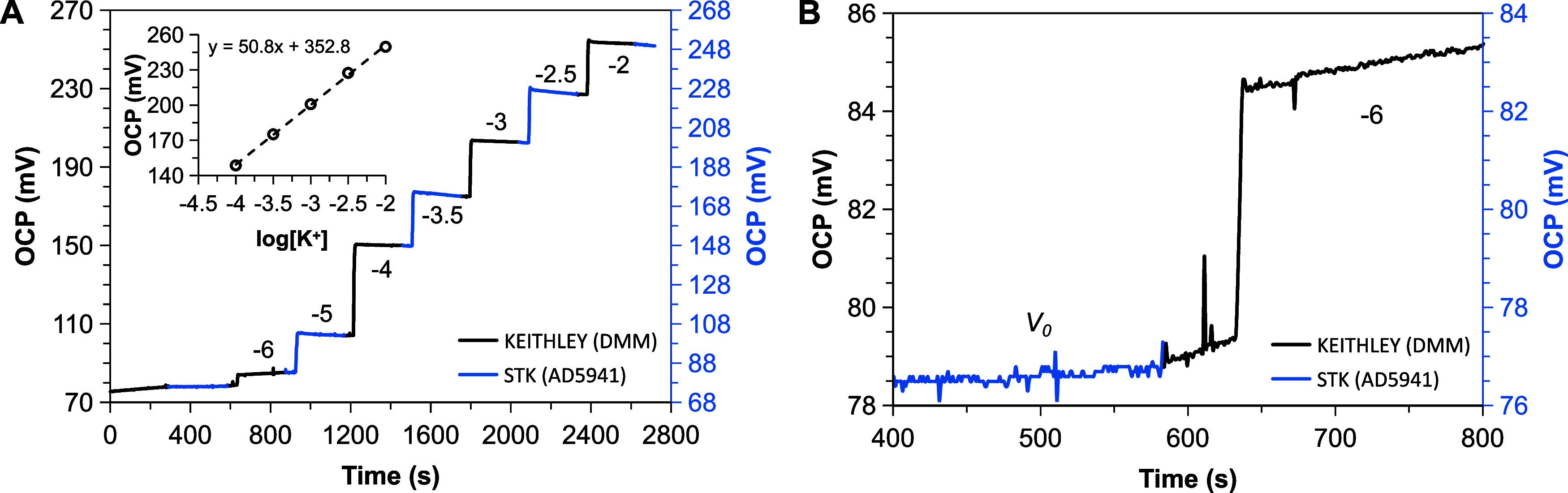
(A) OCP time trace for the detection of K^+^ measured in parallel with the digital electrometer (black line) and STK (blue line), switching between them. The inset shows the corresponding calibration plot. (B) Zoomed-in baseline region showing a maximum offset of 2 mV between the two instruments.

Interdevice reproducibility was also demonstrated for two different STK boards manufactured in the same batch. A consistent sensitivity of 50.2 mV/dec was achieved, with a maximum 0.5 mV signal offset between the two and comparable baseline noise levels well below 1 mV (Figure S5).

The results demonstrate that the STK effectively handles high input impedance signals from ISEs, despite minor signal artifacts attributable to connectors, input switching, and STK–DMM impedance mismatch (Figure 3B).

#### Potentiometric and Self-Current Detection of H_2_O_2_

To further test the input capabilities of the STK board, lower impedance sensors were interfaced (50–100 kΩ). For this purpose, we have selected the recently reported semiopen electrochemical cell (SOEC) that uses a top macroporous working electrode, a polyelectrolyte bridge, and a bottom metallic conductor as an internal reference electrode.^46^ This sensor allows the monitoring of an electrochemical property from an external solution with only the top electrode in direct contact with the sample. The cell offers several advantages such as working in both potentiometric and self-current mode, requiring limited volume of sample and enabling easy multiplexing, i.e., multiple working electrodes bridged with a single reference electrode. First, the open-circuit potential was recorded with STK and the electrometer. As shown in Figure 4A–B, sensitivities of −130 mV/dec were achieved in any case with a logarithmic linearity that spans from 10 to 330 μM. This is in good agreement with our previous works.^46^ The recorded signals showed sub-millivolt offset differences and comparable signal-to-noise ratios (Figure S6). These results confirm the ability of STK to perform stable measurements over a wide range of sensor impedance, stressing the potential of STK as a platform for electrochemical sensing applications.

**Figure 4:**
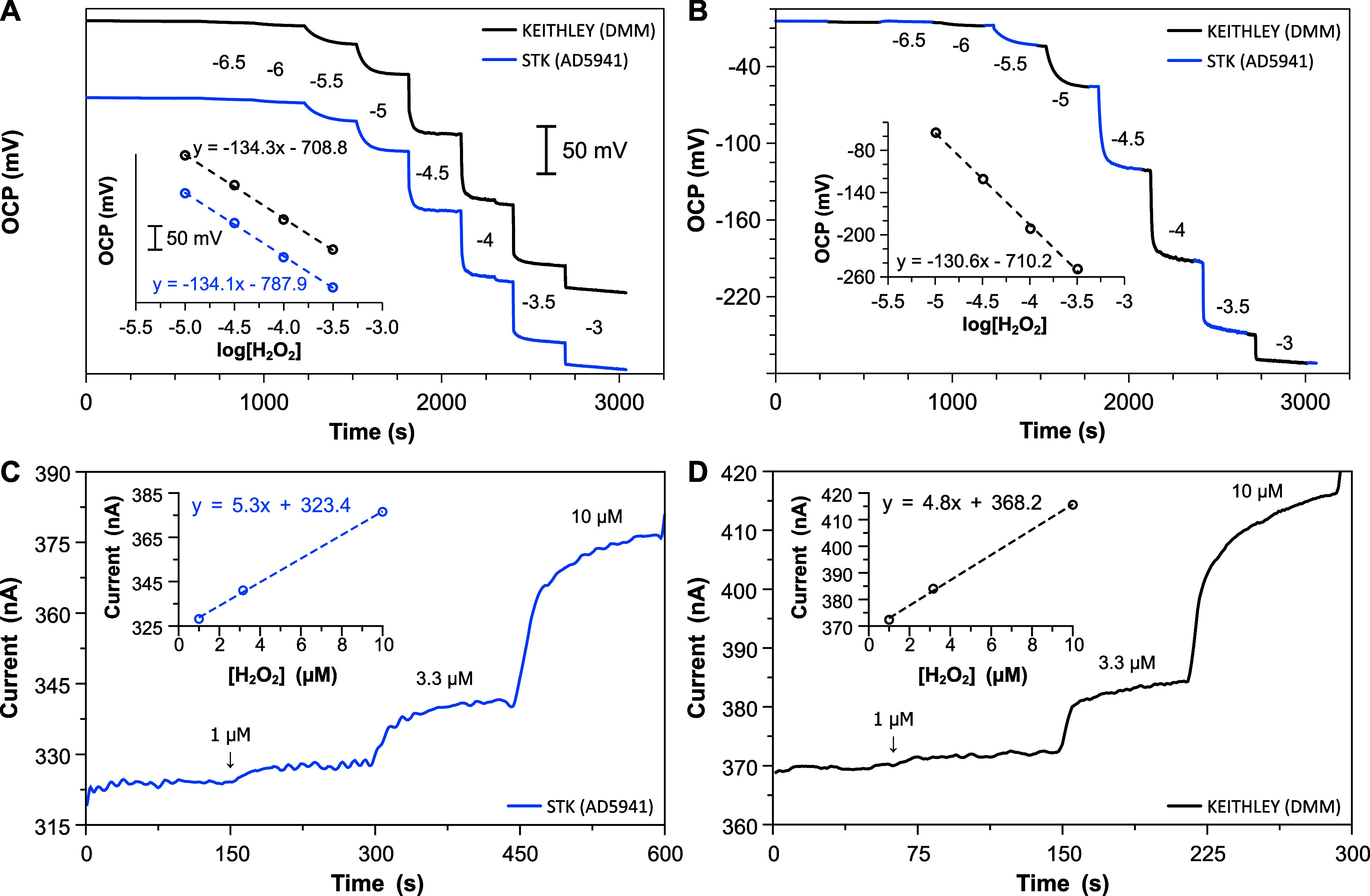
(A, B) OCP time traces for the detection of H_2_O_2_ measured with an electrometer (black line) and STK (blue line): simultaneous readings (A) and under scheduled switching (B). (C, D) Amperometric time trace for the detection of H_2_O_2_ in the lower μM range measured with STK (C) and the electrometer (D) in two different experiments. Data series in (A) are offset by 80 mV—a scale bar corresponding to 50 mV is provided for comparison.

By short-circuiting the working and reference electrodes, the SOEC can also be used in self-power current mode. This current is measured using the transimpedance amplifier nodes of AD5941 in the case of the STK board. Thus, the zero-biased current of the SOEC was monitored upon different concentrations of hydrogen peroxide and validated against the electrometer. A cropped section of the time traces (smoothed by centered moving average, rectangular-weighted) corresponding to the lower μM range is displayed in Figure 4C–D, showing a consistent sensitivity of 5.1 ± 0.5 nA/μM of H_2_O_2_ in the [1, 10] μM range for both cases. Full calibration is provided in Figure S7. Again, both devices afford equal sensor response with comparable analytical performance, demonstrating also the potential of the portable board in current mode applications. Notably, comparable signal-to-noise ratios were obtained despite the compromised amperometric performance of the STK platform (Figure S8).

To validate that the mentioned noise levels were limited by sensor characteristics rather than instrumentation, sensor-independent noise metrics were obtained using precision resistors (Figures S10–S11 and Tables S2–S3). These showcase a noise floor well below the system resolution limit—more than 3-fold lower for typical input signals, based on the electronic limit of detection (3σ)—demonstrating that electronic noise is negligible compared to sensor-related variability dominated by electrochemical processes.

### Input Multiplexing and IoT Hyperconnectivity: Multichannel H_2_O_2_ and Enzyme-Based Detection

To further leverage the on-board capabilities of the STK board and demonstrate its potential in IoT hyperconnected applications, multiplexed sensor readings were sent and fetched remotely via Wi-Fi and a local cloud server mounted on a Raspberry Pi 5. IoT standard MQTT protocol was used for real-time data flow between STK and server-side. Input multiplexing was implemented by using the AD5941 sequencer. Custom SOECs comprising 8 working electrodes and a shared reference electrode were used for the detection of hydrogen peroxide, glucose, and lactate. For the latter, 3 electrodes per analyte were functionalized with immobilized glucose (GOx) and lactate (LOx) oxidases. Two electrodes were left without enzymes as a control.

The OCP of 8 working electrodes was monitored against the common reference when detecting hydrogen peroxide (Figure 5A), as well as glucose and lactate (Figure 5B). Consistent responses were recorded without crosstalk between any of the electrodes, for both enzymatic and nonenzymatic case scenarios. In the case of H_2_O_2_ detection, an average sensitivity of −127 ± 16 mV/dec from 3.3 to 330 μM was obtained with similar time trace shapes for all the electrodes, demonstrating a good reproducibility between them and along the multiplexed inputs. Moreover, the enzymatic sensor exhibited good selectivity, with only the three glucose- and three lactate-specific electrodes responding to either glucose or lactate additions between 0.1 and 10 mM (Figure 5B). A snapshot of the server-side data visualization using Grafana can be seen in Figure 5C and accessed online.^47^

**Figure 5:**
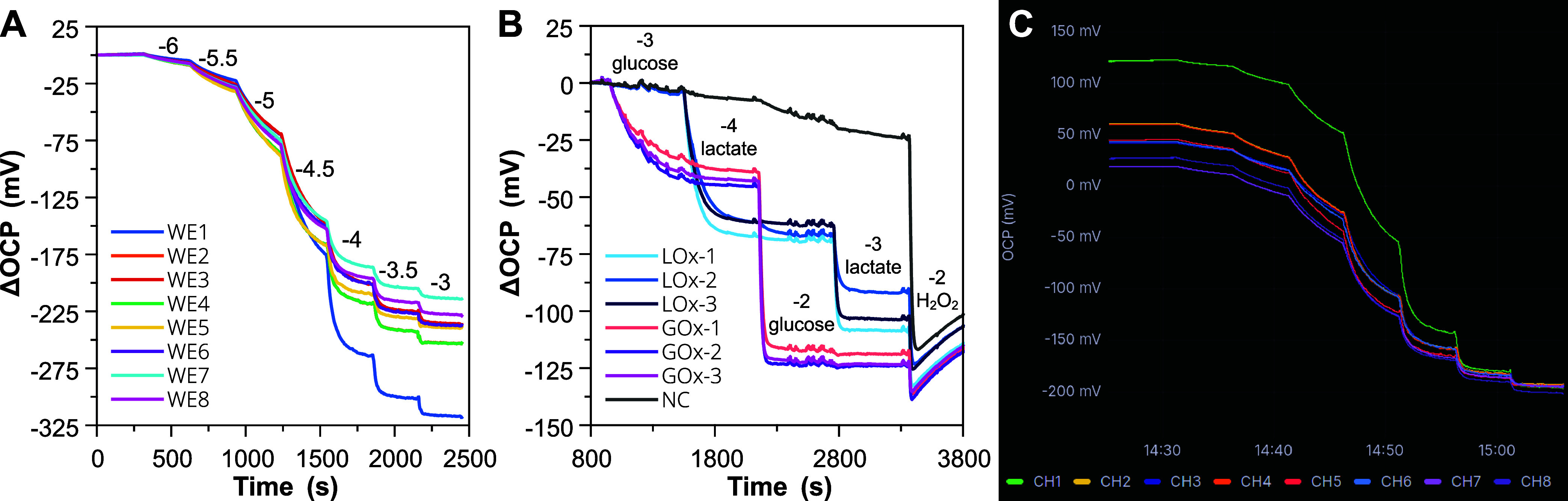
Baseline-subtracted OCP time traces for the detection of H_2_O_2_ (A) and glucose and lactate (B) using STK with an 8-channel multiplexed input configuration. (C) Snapshot of OCP raw data from (A) as visualized in server-side Grafana application. WE: working electrode; LOx: lactate oxidase; GOx: glucose oxidase; NC: control; CH: input channel.

These results again prove that the IoT framework developed for Sensor ToolKit successfully enables an automated data flow from the generation of sensor information toward comprehensive real-time visualization and analysis tools. This provides a platform that is accessible to the end-user, while following open-source standards that minimize knowledge and technological barriers.

Future versions of the Sensor ToolKit smart platform will reduce footprint size, optimize power consumption, and implement the most demanded electrochemical techniques such as EIS, CV, LSV, SWV, and chronoamperometry. The potential extrapolation of sequential multiplexing to amperometric assessments will also be studied, with up to 6 working electrodes sharing a common reference. Eventually, in terms of wireless communication, Bluetooth Low-Energy (BLE), Near-Field Communication (NFC), and ZigBee will be deployed.

## Conclusions

A Sensor ToolKit prototype has been developed and implemented for electrochemical applications with potentiometric and self-current sensors. The associated firmware and IoT framework allow for the deployment of accessible infrastructures well beyond classical potentiostat standards, all assembled into a small, portable, and low-cost device with endless possibilities enabled by open standards.

The design, integrating the AD5941 analog front-end, delivers a performance comparable to most benchtop instrumentation. With excellent signal-to-noise ratios, the proposed device proved to be well suited for analytical applications, where high precision is demanded. Moreover, the multiplexing capabilities are attractive features for the development of PoC and wearable devices.

## Supplementary Material





## Notes

Additional experimental details, materials, and methods, including circuit layouts and schematics, bill of materials, and supporting experimental assessments (PDF)
Sensor ToolKit firmware and server frameworks: STKframework v1.1 (ZIP)
