# Supplementary material for: Sensor ToolKit (STK): Compact Multiplexing Potentiostat for Point-of-Care Applications
Source: Anal Chem. 2026 Feb 17;98(8):5875–82. doi: 10.1021/acs.analchem.5c07176 (PMC12961643; doi:10.1021/acs.analchem.5c07176)
Supplement: Supplementary file 1 [file ac5c07176_si_001.pdf]

Supporting Information

Sensor ToolKit (STK): Compact Multiplexing Potentiostat for Point-of-Care Applications

Andrés Alberto Andreo Acosta[1], Alicia M. Maya[1], Simone Saporito[2], Moritz Kleinstraß[2], Jan Mitrovics[2], Pascal Blondeau[1], Francisco Javier Andrade[1,*]

[1]*Department of Analytical Chemistry, Universitat Rovira i Virgili, Carrer Marcel·lí Domingo, 1, 43007 Tarragona, Spain.*

[2]*JLM Innovation GmbH, Vor dem Kreuzberg 17, 72070 Tübingen, Germany.*

[*]E-mail: franciscojavier.andrade@urv.cat

Number of Pages: 11
Number of Figures: 13
Number of Tables: 7

STK board layout and circuit diagram

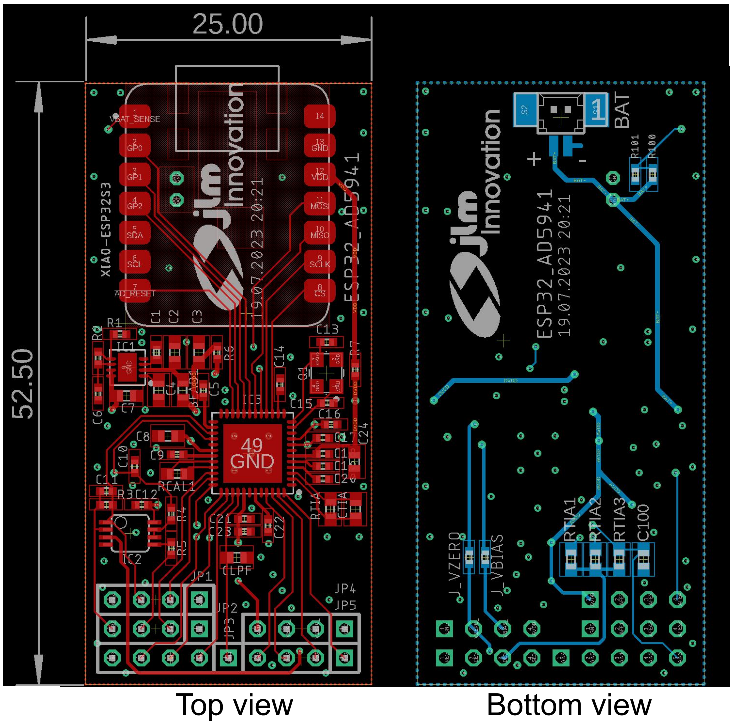

Fig. S1. Top and bottom views of STK printed circuit board design. Dimensions are in millimeters.

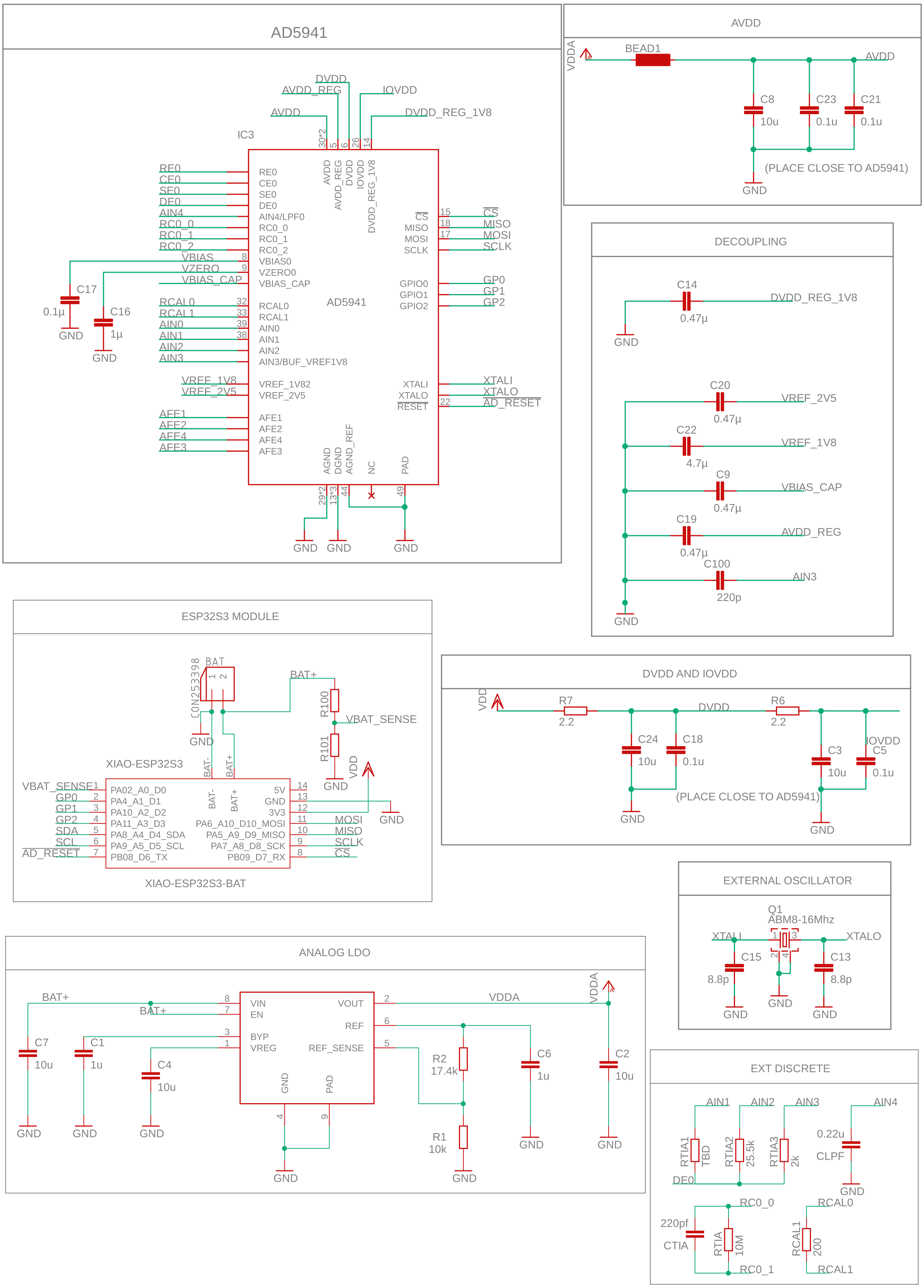

Fig. S2. Circuit schematics of STK main integrated circuits and internal connections.

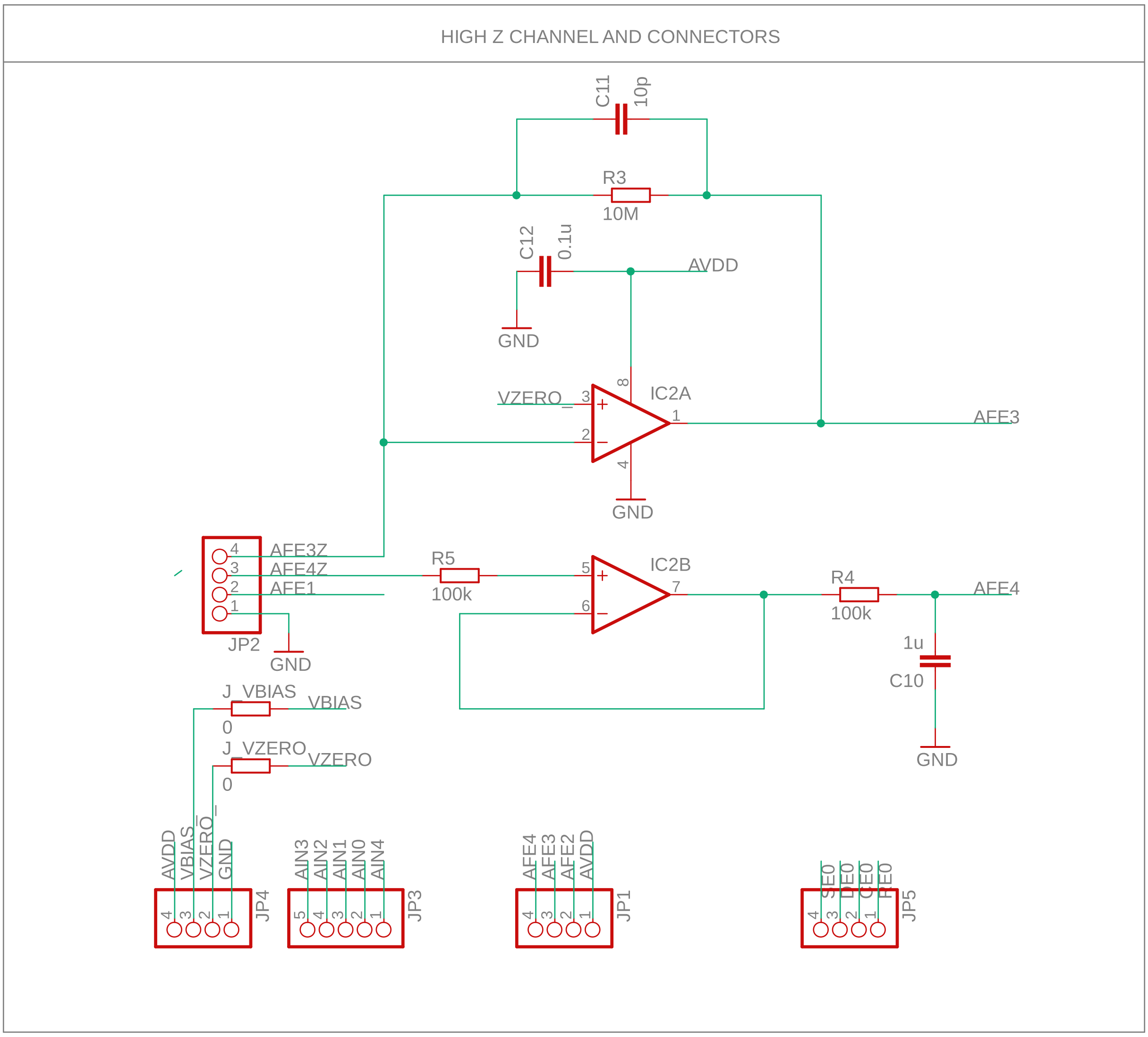

Fig. S3. Circuit schematics of STK high impedance loops and external header pin connections.

Hardware and software frameworks

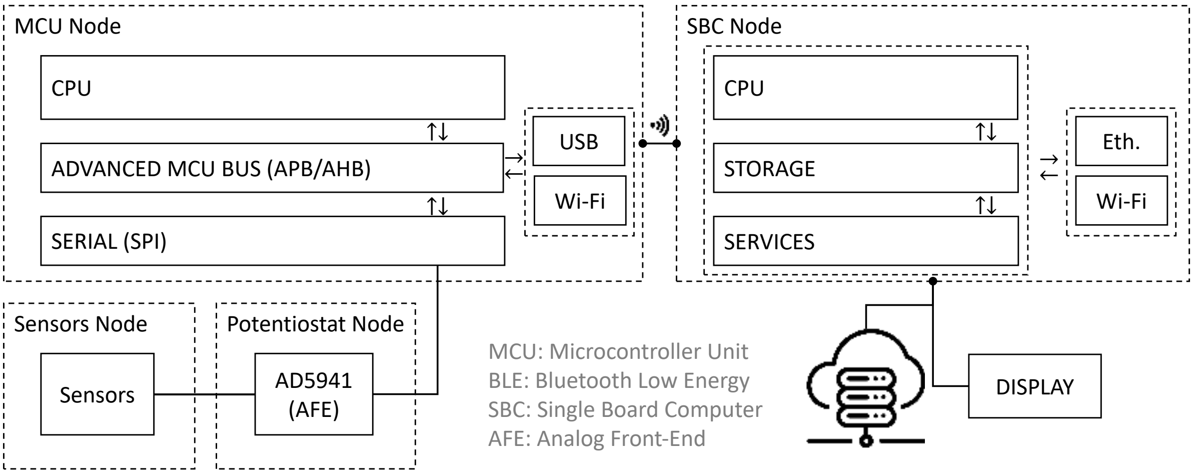

Fig. S4. Block diagram for dataflow from sensor and STK hardware to end-application service or client.

Performance validation and comparison

Inter-device reproducibility

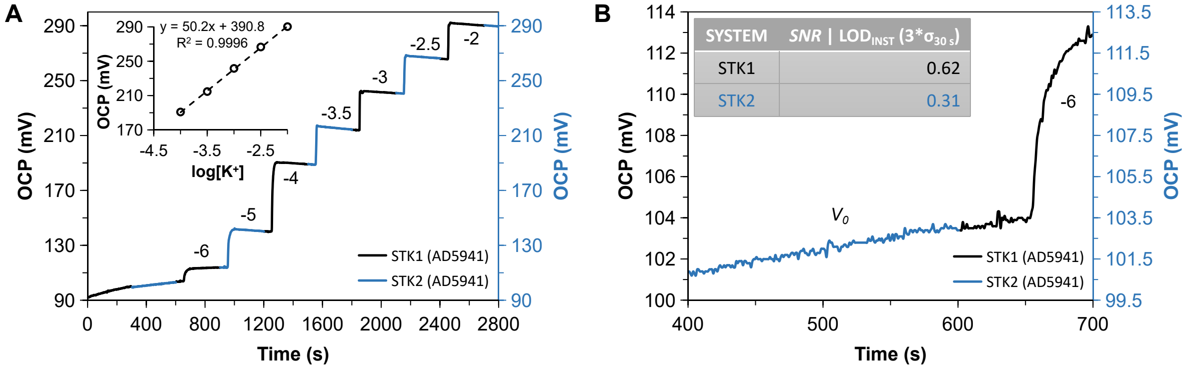

Fig. S5. (A) OCP time trace for the detection of K^+ measured in parallel with two different STK boards of the same manufacturing batch, switching between them. Inset shows the resulting calibration curve. (B) Zoomed-in baseline region comparing the signal-to-noise ratios of the two STK devices and showing a maximum offset of 0.5 mV. SNR was calculated as three times the standard deviation of the signal along a 30-seconds window.

Signal-to-noise ratio and digitation

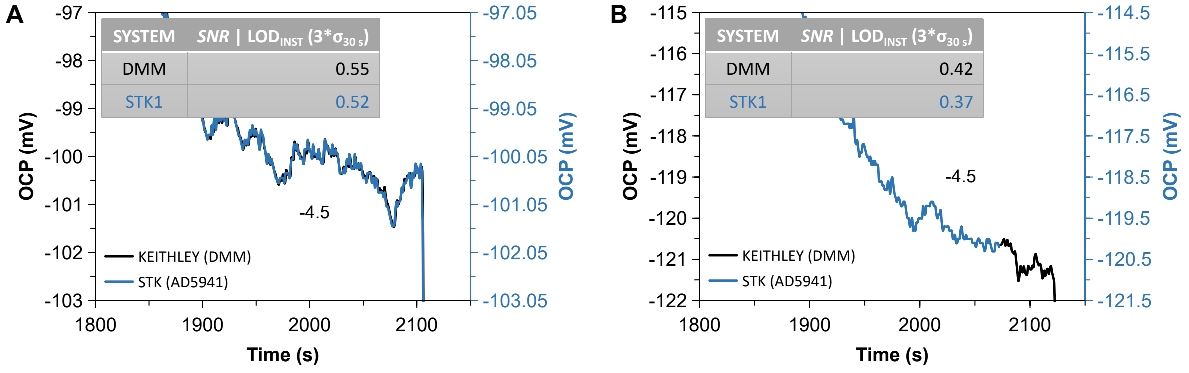

Fig. S6. Zoomed-in OCP time traces for the detection of 33 μM (-4.5, log scale) of H_2O_2 comparing the signal-to-noise ratios of the measurements performed with STK and an electrometer: simultaneous readings (A) and under scheduled switching (A). SNR was calculated as three times the standard deviation of the signal along a 30-seconds window.

Table S1. Performance comparison of SensorToolKit with other potentiostat devices.[a]

Device	V_{IN} range (V)	I_{IN} range	Input CHs (on-board MUX)	Smpl. rate (Hz)[b]	Connectivity	Largest dim. (mm)	Cost (USD)
μSTAT 8000[1]	±4	±1 nA to ±100 mA	8 (8xWE *vs.* 1xRE/CE)	1000	USB, Bluetooth	222	>5000
μSTAT-i 400[2]	±4	±1 nA to ±10 mA	1 (2xWE)	n.d.	USB, Bluetooth	132	>5000
DropStat Plus[3]	±4	±40 mA	1 (2xWE)	n.d.	USB, Bluetooth	130	n.d.
EmStat4 MUX[4]	±3	±1 nA to ±10 mA	8 (8x WE/RE/CE)	1M, ^{M}60	USB	138	>5000
PalmSens4[5]	±10	±100 pA to ±10 mA	1 (–)	150k	USB, Bluetooth	157	>5000
Sensit Wearable[6,7]	−1.7 to 2	±100 nA to ±3 mA	1 (2xWE)	1000	USB, BLE 5.0	35	>1000
Zensor EIWP110[8]	±1.8	±1 nA to ±1 mA	1 (–)	n.d.	USB, 2.4G RF	125	>1000
MicruX EC-Sens[9]	±1.5	±0.25 μA to ±5 mA	2 (2x WE/RE/CE)	n.d.	USB	39	700
SenseItAll[10]	±1	±5 nA to ±1 mA	1 (–)	10	USB, Bluetooth	117	>1700
DStat[11]	±1.46	n.d.	1 (–)	30k	USB	92	120
KickStat[12]	±0.792	±1.5 nA to ±10 mA	1 (–)	60	USB	22	n.d.
Hanitra et al.[13]	0.05 to 0.53	±33 μA	4 (2xOCP + 2xAMP)	300k	USB, BLE 4.0	76	n.d.
Molderez et al.[14]	±10	±100 pA to ±3.3 μA	16 (128xWE)	^{M}651	USB	163	80
SIC434x[15]	±1.2	±2.5 to ±20 μA	1 (–)	10	NFC	85	>80
ACEstat[16]	±1.1	±50 pA to ±3 mA	2 (2x WE/RE/CE)	n.d.	USB	51	60
NanoStat[17]	±0.6	±100 pA to ±10 mA	1 (–)	n.d.	Wi-Fi	40	25
Boni et al.[18]	±1.65	±62 μA	4 (4x WE/RE/CE)	1000	Wi-Fi	∼100	n.d.
OPEN-SENS[19]	±8	±10 nA to ±200 mA	1 (–)	12.5	USB	56	100
FreiStat[20]	±2	±1.8 to ±900 μA	1 (–)	300	USB, Wi-Fi	51	80
SensorToolKit (this work)	±1	±100 nA to ±3 mA	1 (8xWE *vs.* 1xRE/CE)	300, ^{M}30	USB, Wi-Fi[c]	53	50[d]

[a] V_{IN}: input voltage; I_{IN}: input current; CH: channel; MUX: multiplexer; BLE: Bluetooth Low-Energy; RF: radio frequency; OCP: open-circuit potentiometric; AMP: amperometric; WE: working electrode; RE: reference electrode; CE: counter electrode; NFC: near-field Communication.
[b] Typical value. ^{M} indicates *MUX@*.
[c] Easily expandable beyond BLE, NFC, Zigbee, Matter, and LoRaWAN.
[d] Production costs based on bill of materials (Table S7). ∼USD 150 for a commercial product.

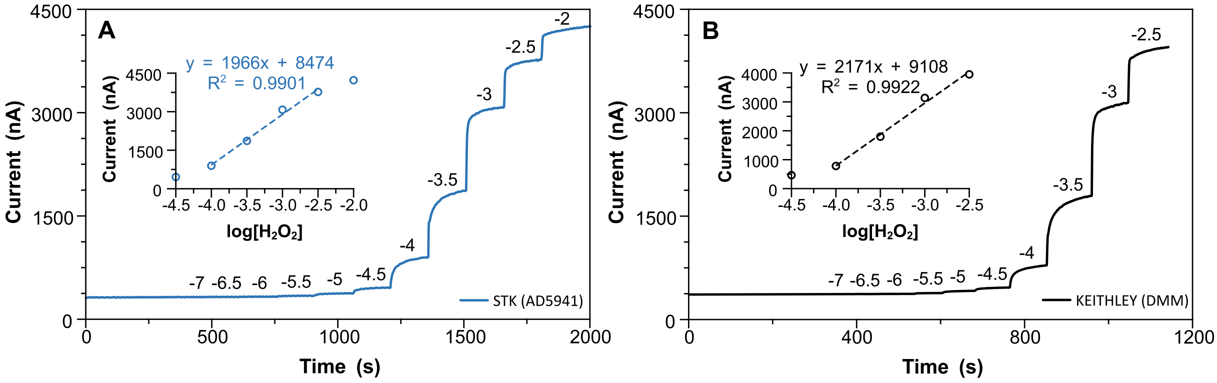

Fig. S7. Amperometric time trace for the detection of H_2O_2 measured with STK (A) and an electrometer (B) in two different experiments. Insets show calibration curves thereof.

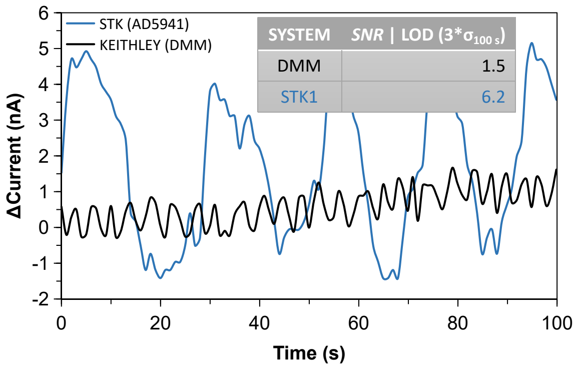

Fig. S8. Zoomed-in baseline of amperometric time traces for the detection of H_2O_2 comparing the signal-to-noise ratios of the measurements performed with STK and an electrometer. SNR was calculated as three times the standard deviation of the signal along a 100-seconds window.

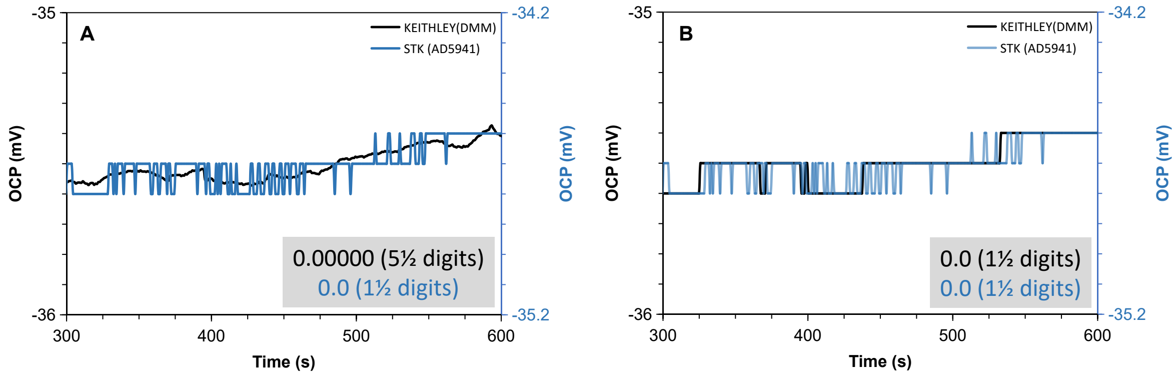

Fig. S9. Zoomed-in baseline of potentiometric time trace for the detection of H_2O_2 comparing the measurement precision as digitized by STK and an electrometer. (A) actual signal readings with $5\frac{1}{2}$ vs. $1\frac{1}{2}$ digits as recorded by the KEITHLEY electrometer and STK, respectively. (B) same signal truncated to $1\frac{1}{2}$ digits for both systems.

Hardware validation: noise floor

For the characterization of the hardware noise floor (Figures S10–S11 and Tables S2–S3), the signal-to-noise ratio (SNR) expressed as the limit of detection (LOD), was calculated as three times the standard deviation (σ) of the signal over a 10-minutes window. Drift values were computed over (at least) a 5-minutes window after reaching 90% of the steady state signal.

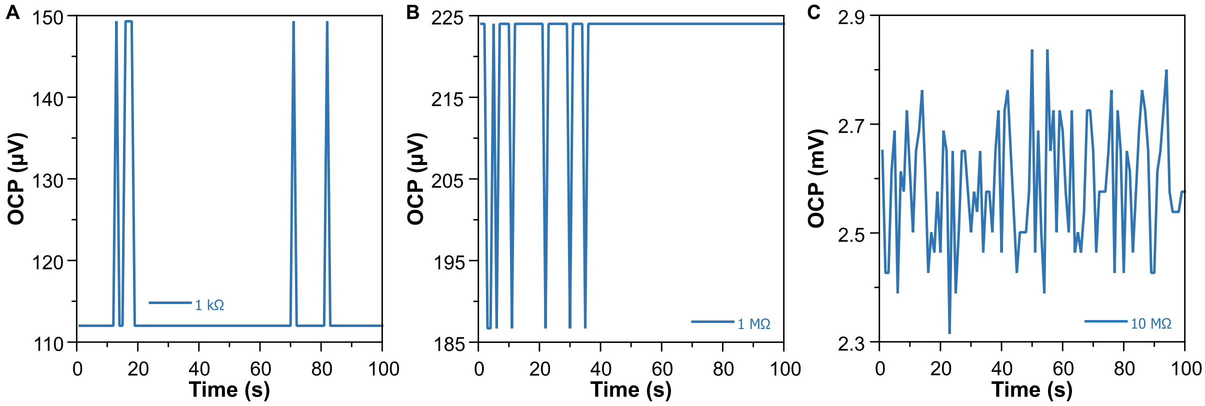

Fig. S10. Zoomed-in baseline noise in OCP measurements using precision resistors of 1 kΩ (A), 1 MΩ (B), and 10 MΩ (C) recorded with STK over 10 minutes.

OCP measurements of pure resistors should deliver signals close to 0 mV. Therefore, mean offset values are provided in Table S2, showing a low noise dominated by the on-chip analog-to-digital converter for lower-value resistors. Higher variation was recorded for 10 MΩ due to the input bias current and leakage limitations of the front-end. Typical input impedance values for OCP range from 1 kΩ to 1 MΩ.

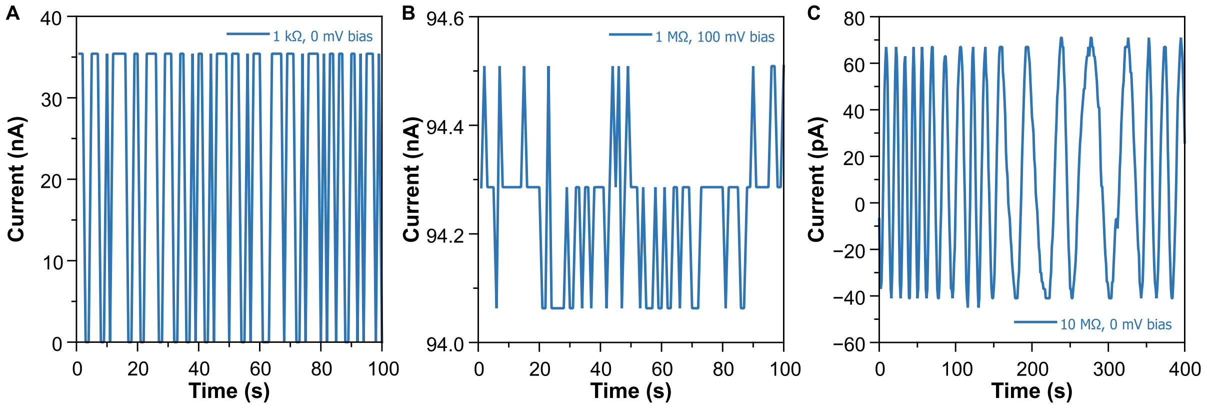

Fig. S11. Zoomed-in baseline noise in amperometric measurements using precision resistors of 1 kΩ (A), 1 MΩ (B), and 10 MΩ (C) recorded with STK over 10 minutes at 0, 100, and 0 mV, respectively.

The amperometric measurements (Figure S11 and Table S3) accounted for the AD594x maximum input current ratings[21]. Thus, the input impedance range expanded from 10 kΩ to 10 MΩ, although electrochemical noise ultimately hindered the upper limit. A voltage bias of either 0 or 100 mV was set between the working and reference inputs for the amperometric validation. Counter electrode was internally shorted to RE. Measurements with zero-biased resistors were performed when suitable bias conflicted maximum input current ratings. Comparable noise level was recorded for the typical 100 kΩ–1 MΩ input impedance range, validating the electronic noise floor of the system against that of electrochemical processes.

Table S2. Sensor ToolKit noise floor characterization using precision resistors in OCP mode.[a]

Resistor	Mean offset $\pm$ σ (μV)	LOD | 3σ (μV)	Drift (μV/min)
1 kΩ	115 ± 9	28	0.69
10 kΩ	113 ± 6	18	0.05
1 MΩ	224 ± 3	10	2.20
10 MΩ	2514 ± 129	387	32.13

[a] σ: standard deviation; LOD: limit of detection.

Table S3. Sensor ToolKit noise floor characterization using precision resistors in amperometric mode.[a]

Resistor	Bias (mV)	Avg. signal $\pm$ σ	LOD | 3σ	Drift (pA/min)
1 kΩ	0	25 ± 18 nA	53 nA	745.3
100 kΩ	0	82 ± 108 pA	323 pA	1.5
100 kΩ	100	920.80 ± 0.14 nA	0.42 nA	7.6
1 MΩ	0	-253 ± 89 pA	266 pA	1.1
1 MΩ	100	94.23 ± 0.13 nA	0.39 nA	1.5
10 MΩ	0	8 ± 38 pA	112 pA	1.6

[a] σ: standard deviation; LOD: limit of detection.

Analytical and sensor-to-device performances

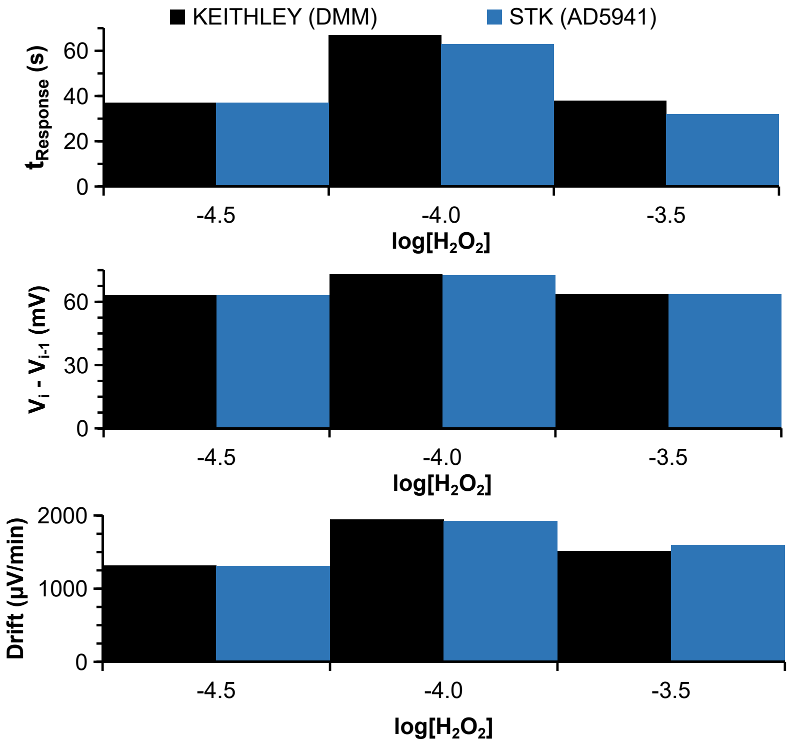

Fig. S12. Comparison of sensor potentiometric performance and kinetics for the detection of H_2O_2 with STK and an electrometer expressed as response time (top), total response (mid), and drift (bottom) upon additions of 33 μM (-4.5, log scale), 100 μM (-4), and 330 μM (-3.5) of H_2O_2. Results show no significant differences between the two compared devices.

Table S4. Analytical performance of different potentiometric sensors assessed in this work.[a]

Sensor	Sensitivity ± σ (mV/dec)	LR	N
K^+ ISE	50.5 ± 0.8	[0.1, 10] mM	4
H_2O_2 SOEC	-127 ± 16	[3.3, 330] μM	16

[a] σ: std. deviation; LR: linear range; N: no. of devices; ISE: ion-selective electrode; SOEC: semi-open electrochemical cell[22].

Table S5. Analytical performance of the H_2O_2 SOEC sensor in self-current mode.[a]

Sensor	Sensitivity ± σ (nA/μM)	LR (mM)	N
H_2O_2 SOEC	5.1 ± 0.5	[1, 10]	2

[a] SOEC: semi-open electrochemical cell[22]; σ: std. deviation; LR: linear range; N: no. of devices.

Table S6. Analytical performance of the multiplexed SOEC enzymatic sensors.[a]

[Analyte] (mM)	OCP ± σ (mV)	N
0.1 (lactate)	-61 ± 4	3
1 (lactate)	-35 ± 9	3
1 (glucose)	-38 ± 5	3
10 (glucose)	-80.4 ± 0.7	3

[a] SOEC: semi-open electrochemical cell[22]; σ: std. deviation; OCP: open circuit potential; N: no. of devices.

OCP input multiplexing

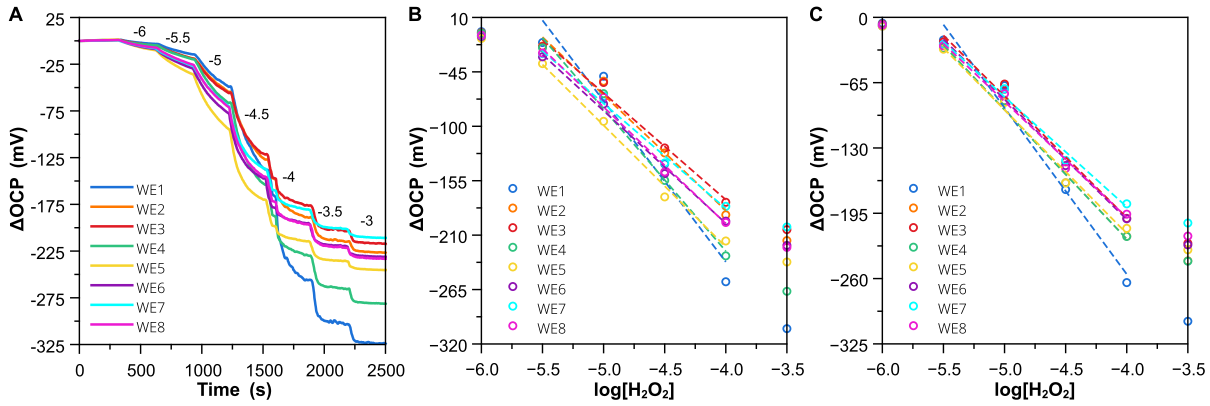

Fig. S13. Baseline-subtracted OCP time traces for the detection of H_2O_2 (A) and calibration curves (B) using a Lawson high input impedance multiplexed electrode interface. (C) Calibration curves for the detection of H_2O_2 using STK with an 8-channel multiplexed input configuration (baseline-subtracted).

Bill of materials for the STK board

Table S7. Bill of materials for the Sensor ToolKit board.[a]

Board part designator(s)	Description	Supplier	Unit price (USD)	Qty.	Total (USD)
BAT	CON253398 (Battery Connector)	Farnell	0.6	1	0.6
BEAD1	MLZ1608M100WT000 (Inductor)	Farnell	0.11	1	0.11
C1, C6, C10, C16	1 μF 0402 Ceramic Capacitor	Mouser	0.08	4	0.32
C100, CTIA	220 pF 0603 Ceramic Capacitor	Mouser	0.37	2	0.74
C11	10 pF 0402 Ceramic Capacitor	Mouser	0.08	1	0.08
C13, C15	8.8 pF 0402 Ceramic Capacitor	Mouser	0.17	2	0.34
C2, C3, C4, C7, C8, C24	10 μF 0603 Ceramic Capacitor	Mouser	0.15	6	0.9
C22	4.7 μF 0402 Ceramic Capacitor	Mouser	0.15	1	0.15
C5, C12, C17, C18, C21, C23	100 nF 0402 Ceramic Capacitor	Mouser	0.06	6	0.36
C9, C14, C19, C20	470 nF 0402 Ceramic Capacitor	Mouser	0.21	4	0.84
CLPF	220 nF 0603 Ceramic Capacitor	Mouser	0.12	1	0.12
IC1	ADM7155ACPZ-04 (LDO)	Farnell	3.48	1	3.48
IC2	LTC6078IMS8 (Dual op-amp)	Mouser	7.68	1	7.68
IC3	AD5941BCPZ (AFE)	Mouser	15.74	1	15.74
JP1, JP2, JP4, JP5	Pin Header 1x4 (2.54mm)	Mouser	0.1	4	0.4
JP3	Pin Header 1x5 (2.54mm)	Mouser	0.12	1	0.12
Q1	ABM8-9-B1U-TABM8 (XTAL)	Mouser	0.64	1	0.64
R1	10 kΩ 0402 Resistor (1%)	Farnell	0.01	1	0.01
R100, R101	22 kΩ 0402 Resistor (1%)	Farnell	0.01	2	0.02
R2	17.4 kΩ 0402 Resistor (1%)	Farnell	0.01	1	0.01
R3	10 MΩ 0402 Resistor (1%)	Farnell	0.01	1	0.01
R4, R5	100 kΩ 0402 Resistor (1%)	Farnell	0.01	2	0.02
R6, R7	2.2 Ω 0402 Resistor (1%)	Farnell	0.01	2	0.02
RCAL1	200 Ω 0603 Resistor (0.1%)	Farnell	0.07	1	0.07
RTIA	10 MΩ 0603 Resistor (0.1%)	Farnell	0.02	1	0.02
RTIA2	25.5 kΩ 0603 Resistor (0.1%)	Farnell	0.2	1	0.2
RTIA3	2 kΩ 0603 Resistor (0.1%)	Farnell	0.13	1	0.13
XIAO-ESP32S3	XIAO ESP32-S3 (MCU)	Mouser	7.49	1	7.49
	Printed Circuit Board (PCB)	Beta Layout	5	1	5
					**45.62**

[a] LDO: low-dropout voltage regulator; op-amp: operational amplifier; AFE: analog front-end; XTAL: crystal oscillator; MCU: microcontroller unit; PCB: printed circuit board.

Reagents and materials

Ion-selective membrane. A potassium ionselective membrane (ISM) contains 2 wt % (18 mmol/kg) of valinomycin, 0.5 wt % (10 mmol/kg) of potassium tetrakis (4-chlorophenyl) borate (KTFPB), 32.8 wt % of poly(vinyl chloride) (PVC), and 64.7 wt % of bis(2-ethylhexyl) sebacate (DOS).

References

[1] µSTAT 8000 brochure. Metrohm DropSens, S.L.U.: Spain, 2025; DS18000011.

[2] µSTAT-i 400 brochure. Metrohm DropSens, S.L.U.: Spain, 2025; DS14004021.

[3] DropSens Plus brochure. Metrohm DropSens, S.L.U.: Spain, 2025; 80005462EN.

[4] EmStat4 MUX brochure. PalmSens BV: The Netherlands, 2025; Rev. 5-2025-001.

[5] PalmSens4 brochure. PalmSens BV: The Netherlands, 2025; Rev. 3-2025-019.

[6] EmStat Pico datasheet. PalmSens BV: The Netherlands, 2023; Rev. 7-2023-011.

[7] Sensit Wearable brochure. PalmSens BV: The Netherlands, 2025; Rev. 02-2025-008.

[8] Zensor R&D co.,Ltd Zensor EIWP110 product website. 2025; https://www.zensorrd.com/EIWP100.html, Accessed: 2025-03-12.

[9] MicruX ECSens bipot brochure. MicruX Technologies: Spain, 2024.

[10] SenseItAll brochure. Zimmer & Peacock AS: Norway, 2025; Rev. 0.

[11] Dryden, M. D. M.; Wheeler, A. R. DStat: A Versatile, Open-Source Potentiostat for Electroanalysis and Integration. *PLOS ONE* **2015**, *10*, e0140349.

[12] Hoilett, O. S.; Walker, J. F.; Balash, B. M.; Jaras, N. J.; Boppana, S.; Linnes, J. C. KickStat: A Coin-Sized Potentiostat for High-Resolution Electrochemical Analysis. *Sensors* **2020**, *20*, 2407.

[13] Hanitra, I. N.; Criscuolo, F.; Pankratova, N.; Carrara, S.; Micheli, G. D. Multichannel Front-End for Electrochemical Sensing of Metabolites, Drugs, and Electrolytes. *IEEE Sensors Journal* **2020**, *20*, 3636–3645.

[14] Molderez, T. R.; Rabaey, K.; Verhelst, M. A Scalable 128-Channel, Time-Multiplexed Potentiostat for Parallel Electrochemical Experiments. *IEEE Transactions on Circuits and Systems I: Regular Papers* **2021**, *68*, 1068–1079.

[15] Krorakai, K.; Klangphukhiew, S.; Kulchat, S.; Patramanon, R. Smartphone-Based NFC Potentiostat for Wireless Electrochemical Sensing. *Applied Sciences* **2021**, *11*, 392.

[16] Brown, E. W.; Glasscott, M. W.; Conley, K.; Barr, J.; Ray, J. D.; Moores, L. C.; Netchaev, A. ACEstat: A DIY Guide to Unlocking the Potential of Integrated Circuit Potentiostats for Open-Source Electrochemical Analysis. *Analytical Chemistry* **2022**, *94*, 4906–4912.

[17] Lee, S. C.-H.; Burke, P. J. NanoStat: An open source, fully wireless potentiostat. *Electrochimica Acta* **2022**, *422*, 140481.

[18] Boni, A.; Bianchi, V.; Fortunati, S.; Giannetto, M.; Careri, M.; De Munari, I. A stand-alone portable potentiostat with parallel channels for smart electrochemical analyses. *IEEE Transactions on Instrumentation and Measurement* **2022**, 1–1.

[19] Trinh, V. D.; Le, M. K.; Le, P. L.; Ho, A. T.; Nguyen, H. A.; Thai, T. T.; Hoang, T. B. T.; Nguyen, T. T. H.; Huynh, L. T. N.; Huynh, D. C. OPENSENS: a low-cost and multi-purpose electrochemical platform. *Journal of Applied Electrochemistry* **2023**, *54*, 783–789.

[20] Bill, D.; Jasper, M.; Weltin, A.; Urban, G. A.; Rupitsch, S. J.; Kieninger, J. Electrochemical Methods in the Cloud: FreiStat, an IoT-Enabled Embedded Potentiostat. *Analytical Chemistry* **2023**, *95*, 13003–13009.

[21] AD5941 datasheet. Analog Devices, Inc.: MA, USA, 2022; Rev. C.

[22] Clua Estivill, M.; Baez, J. F.; Blondeau, P.; Andrade, F. J. Electrochemical Pixels: Semi-open electrochemical cells with a vertically stacked design. *Biosensors and Bioelectronics* **2024**, *246*, 115877.
