# Supplementary figures and images for: Sensor ToolKit (STK): Compact Multiplexing Potentiostat for Point-of-Care Applications
Source: Anal Chem. 2026 Feb 17;98(8):5875–82. doi: 10.1021/acs.analchem.5c07176 (PMC12961643; doi:10.1021/acs.analchem.5c07176)

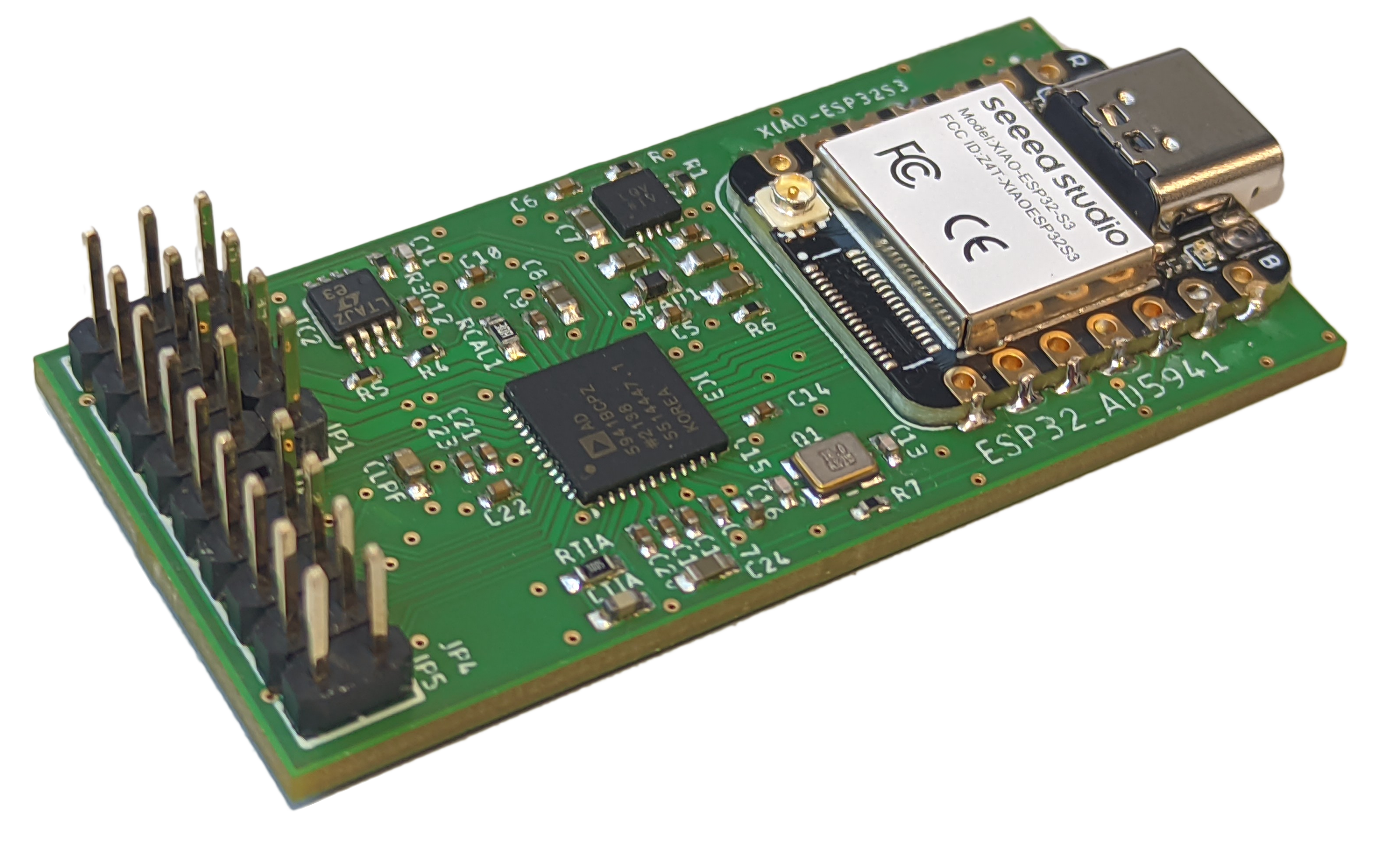

Supplement: Supplementary file 2 [file ac5c07176_si_002.zip › SensorToolKit-STK-main/STK.png]
